# Monte Carlo Simulation of TRIM Algorithm in Ceramic Biomaterial in Proton Therapy

**DOI:** 10.3390/ma16134833

**Published:** 2023-07-05

**Authors:** Fatih Ekinci, Tunc Asuroglu, Koray Acici

**Affiliations:** 1 Institute of Nuclear Sciences, Ankara University, 06830 Ankara, Turkey; fatihekinci@ankara.edu.tr; 2 Faculty of Medicine and Health Technology, Tampere University, 33720 Tampere, Finland; 3 Artifical Intelligence and Data Engineerig, Ankara University, 06830 Ankara, Turkey; kacici@ankara.edu.tr

**Keywords:** bioceramics, Monte Carlo, TRIM algorithm, zirconia, medicine

## Abstract

Biomaterials play a crucial role in enhancing human health and quality of life. They are employed in applications such as tissue substitution, diagnostic tools, medical supplies, therapeutic treatments, regenerative medicine, and radiation dosimetric studies. However, their predisposition to proton therapy, which is a trending treatment in the world, has not been adequately studied. Ceramic biomaterials, known for their hardness and durability, offer versatile uses, especially in bone tissue replacements. The wide range of physical, mechanical, and chemical properties exhibited by ceramics has spurred extensive research, development, and application in this field. This study focuses on investigating and analyzing the ionization, recoils, phonon release, collision events, and lateral scattering properties of ceramic biomaterials that closely resemble bone tissue in proton therapy applications. Monte Carlo (MC) Transport of Ions in Matter (TRIM) simulation tools were utilized for this analysis. The results showed that Silicon dioxide exhibited the Bragg peak position closest to bone tissue, with a deviation of 10.6%. The average recoils differed by 1.7%, and the lateral scattering differed by 3.6%. The main innovation of this study lies in considering interactions such as recoil, collision events, phonon production, and lateral scattering when selecting biomaterials, despite their limited digitization and understanding. By evaluating all these interactions, the study aimed to identify the most suitable ceramic biomaterial to replace bone tissue in proton therapy.

## 1. Introduction

Proton therapy is thought to have greater potential to preserve healthy tissue than conventional photon therapy [1]. Providing overdose to deep-seated or radiation-resistant tumors and less radiation to the healthy tissue around the tumor are the most important advantages of this approach [2,3]. This provides most of the energy of a single-energy proton beam, leaving the peak Linear Energy Transfer (LET) depth within a narrow depth range known as the Bragg peak [4]. Due to the narrow depth range of the Bragg peak, it delivers a therapeutic dose that is highly compatible with the target volume, with minimal lateral scattering [5,6] and an input dose much lower than is possible with photon therapy [7]. The success of proton therapy depends on the correct LET measurement and the accuracy of the LET value obtained with the help of semi-analytical pencil beam algorithms [8].

The accuracy of the LET calculation algorithm in proton therapy is critical in terms of taking full advantage of the ballistic (focus on target) potential of protons. Monte Carlo (MC) proton transport simulation is considered the most accurate approach [9,10]. The expected medical benefit of MC simulation is better to control the administered doses with a reduction in treatment toxicity due to the proton therapy margin reduction [11]. This control is performed by using phantoms. It is generally accepted that these phantoms give accurate results in measurements and that the phantom structure is related to tissue equivalence [12].

Bioceramics used as phantoms can be of synthetic or natural origin, designed to bond with bone, and emerged as an alternative to metallic implants [13,14,15]. They are important in the field of biomedicine because of their good and compatible physico-chemical properties with certain parts of the human body [16,17,18]. Ceramic applications, especially in the 20th century, have been widely preferred in the field of medicine due to the advances in processing technology [19,20,21]. They are preferred over metal-based biomaterials because of their excellent biocompatibility, poor degradability, high melting temperature, non-corrosive, better mechanical properties, and poor plasticity [16]. Bioceramics are hard and brittle and have low fracture toughness with elastic modulus compared to bone [12,22,23]. Synthetic bioceramics such as alumina, zirconia, titania, and bioactive glasses/glass ceramics are used in dentistry, orthopedics, calcified tissues, implants, coatings, medical sensors, and many other applications [24]. The use of bioceramics also provides a new horizon in the context of hard tissue repair and regeneration [25]. The main innovation of this study lies in considering interactions such as recoil, collision events, phonon production, and lateral scattering when selecting biomaterials, despite their limited digitization and understanding. By evaluating all these interactions, the study aimed to identify the most suitable ceramic biomaterial to replace bone tissue in proton therapy.

## 2. Material and Method

Ceramic materials used as biomaterials in this study, namely, alumina (Al_2_O_3_), silicon dioxide (SiO_2_), titanium dioxide (TiO_2_), and zirconium dioxide (ZrO_2_) were simulated by MC TRIM to be compared with bone tissue. Mass densities (g/cm^3^), atomic number density (×10^22^ atoms/cm^3^), and basic chemical compositions in percent (%) of ceramic biomaterials are given in Table 1. The TRIM simulation program determined these percentages according to the ICRU-276 report [26].

**Figure 1:**
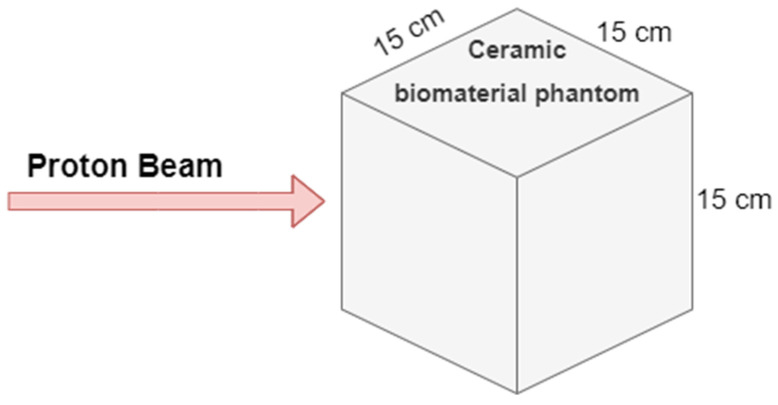
In the MC TRIM system, the figure shows the shape and size of the phantom formed from the ceramic biomaterial and the direction of progression of the proton beam.

The phantom in Figure 1 was formed from these ceramic biomaterials in a volume of 15 × 15 × 15 and was bombarded with a pencil beam of 10⁶ protons at therapeutic energies (60, 80, 100, 120, 140, and 160 MeV). The energy employed in this study is set at the average therapeutic energy level and was administered in increments of 20 MeV on average. The purpose of these increments is to facilitate progress in material scanning, covering an average area below 2 cm. To obtain the desired calculation parameters, the average application time at each energy level was considered through the use of the TRIM simulation program. These calculation parameters are determined by TRIM, incorporating probability calculations due to the nature of MC simulations.

The MC TRIM simulation system used in this study can calculate all interactions of the proton beam inside the phantom [27]. The energy of the proton beam, the number of particles, the selected phantom type and geometry and the parameters to be calculated can be entered from the MC TRIM screen [27]. MC TRIM can calculate all kinetic events related to energy dissipation processes of the proton beam, such as damage, scattering, ionization, voids in the crystal structure of ceramic biomaterials, phonon production, and recoil in the selected ceramic phantom [27]. Furthermore, it can track and record the target atomic cascades in selected ceramic phantoms in detail [28].

The important innovations provided in this study are the parameters of recoils and collision events. These parameters are expressed by the Kinchin–Pease theory. It is used to calculate the displacements of proton beams and atoms of ceramic biomaterials [29]. The displacement with the Norgett, Robinson, and Torrens [30] model is used to calculate the number of primary impact atoms. Number of displaced atoms *N_v_*, Norgett, Robinson, and Torrens model:

(1) $$N_{v}\left\{\begin{array}{cc} 0 & E_{v}\;<\;E_{d}\\ 1 & E_{d}\leq E_{v}\;<\;2.5E_{d}\\ \frac{0.8\;E_{v}}{2\;E_{d}} & E_{v}\geq 2.5E_{d} \end{array}\right\}$$

In Equation (1), while *E_v_* refers the damage energy, *E_d_* is for the threshold displacement energy [29]. Recoils and collision events parameters are calculated with the help of Equation (1).

The other concept that this study focuses on is the lateral scattering parameter. This parameter *x_i_* is the projection range of the *“i”* ion on the *x*-axis; Σ*_i_ x_i_* = sum of ion projection ranges; Σ*_i_ x_i_/N* = mean projection range of *N* ions and <*x*> = mean projection range of all ions [28]. Considering the transverse coordinate *“y”* in the same way, only the distance in the *XY* plane is considered [28]. Therefore, the lateral scattering is:

σ = [(Σ*_i_ x_i_*^2^)/*N* − R_p_^2^]^1/2^ = <(Δ*x_i_*)^2^>^1/2^ (2)

It is defined as given in Equation (2). For a normal sent projectile proton beam, the cylindrical symmetry of the gap distribution can be assumed, so the average lateral reflected range is zero (i.e., *Ry* = 0) [13]. Moreover, the ranges predicted by *Y* and *Z* are averaged to increase the accuracy of the calculation [28]. Therefore, the lateral scattering is expressed as given in Equation (3):

σ*_y_* = [Σ*_i_* ((|*y_i_*| + |*z_i_*|)/2)^2^/*N*]^1/2^ (3)

## 3. Results

### 3.1. Bragg Cure

The Bragg peak position and amplitude formed (electronvolt (eV) and Angstrom (A)) in Al_2_O_3_, SiO_2_, TiO_2_, ZrO_2,_ and bone phantoms of the proton beam with 60–160 MeV energy obtained from this study are shown in Table 2. For every 20 MeV energy increase in the proton beam, the Bragg peak range increased 29.5% in Al_2_O_3_, 28.7% in bone, 28.5% in SiO_2_, 27.7% in TiO_2,_ and 27.2% in ZrO_2_. The Bragg peak range is average, respectively, for the ceramic biomaterial closest to the bone; SiO_2_ with a difference of 10.6%, TiO_2_ with a difference of 45.2%, Al_2_O_3_ with a difference of 46.9%, and ZrO_2_ with a difference of 53.6% were obtained. When Bragg peak amplitude was compared with bone, the closest ceramic biomaterial value was formed in SiO_2_ biomaterial with an average difference of 3.2%. In other biomaterials, the average differences are, respectively, TiO_2_ 45.2%, Al_2_O_3_ 46.9%, and ZrO_2_ 53.6%.

**Figure 2:**
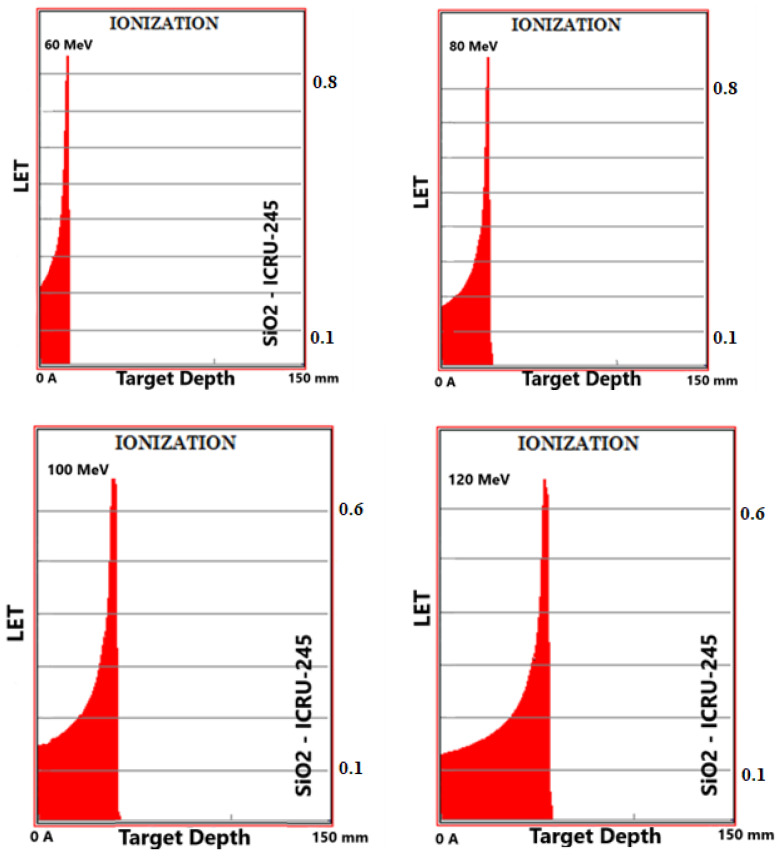

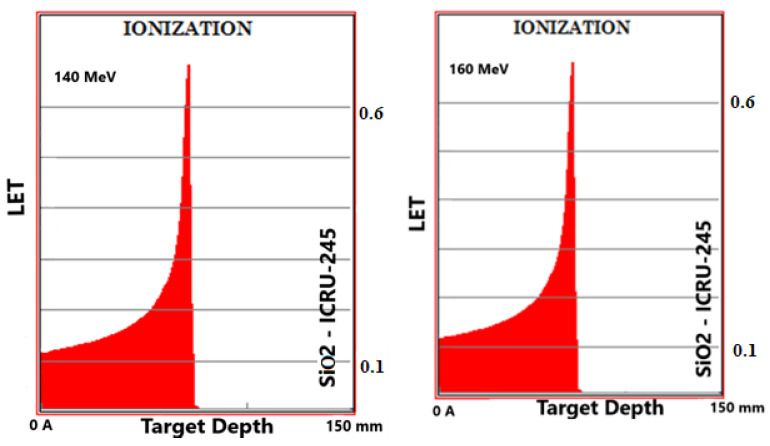
MC TRIM simulation output figures of ionization (LET (ev/A)) values in SiO_2_ ceramic biomaterial of a 106-particle proton beam with 60–160 MeV energy.

Simulation outputs of SiO_2_ biomaterial, which is the ceramic biomaterial that gives the closest value to hard tissue in ionization results are given in Figure 2.

### 3.2. Recoils

The recoils peak (eV/A-ion) formed by the proton beam in selected ceramic and bone phantoms and the percentage contribution of the atoms forming the phantoms to this peak are given in Table 3. The average recoil value of bone tissue in six energy ranges was 0.2697 eV/A-ion. The average contribution of atoms to these average recoils value is, respectively. It is composed of 27.2% H, 22.4% C, 32.7% O, 2.2% N, 0.2% Mg, 4.9% P, 0.2% S, and 10.2% Ca. The average recoil value of the Al2O3 phantom in six energy ranges was 0.345 eV/A-ion. The contribution of atoms to these average recoils value is, respectively, composed of 49.1% Al and 50.9% O. The average recoil values of SiO_2_, TiO_2,_ and ZrO_2_ phantoms in six energy ranges were 0.265, 0.352, and 0.346 eV/A-ion, respectively. The contribution of atoms to these average recoils value consisted of 46.5% Si and 53.5% O in the SiO_2_ phantom, 52.5% Ti and 47.5% O in the TiO_2_ phantom, and 63.9% Z and 36.1% O in the ZrO_2_ phantom.

**Figure 3:**
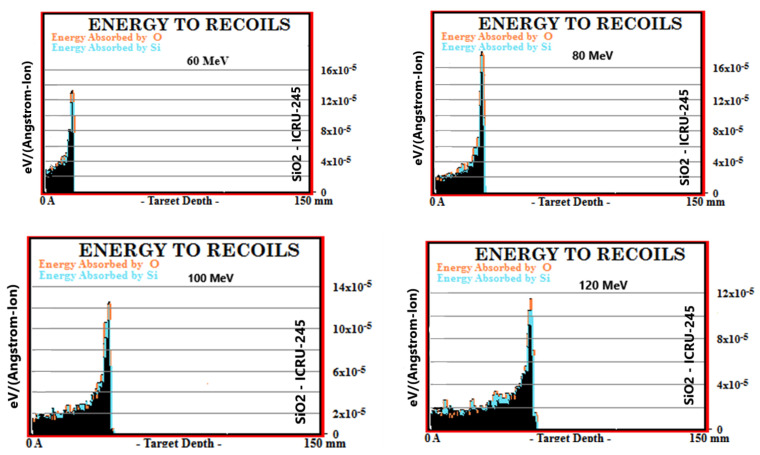

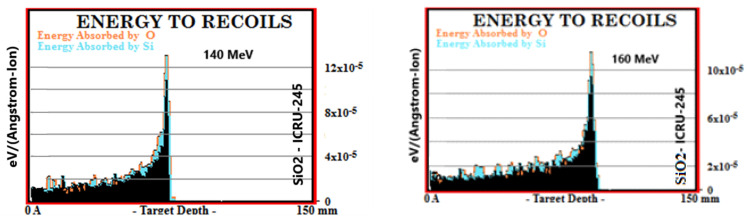
MC TRIM simulation output figures of recoils values in SiO_2_ ceramic biomaterial of a 106-particle proton beam with 60–160 MeV energy.

Simulation outputs of SiO_2_ biomaterial, which is the ceramic biomaterial that gives the closest value to hard tissue in recoil results are given in Figure 3.

### 3.3. Phonon Production

The phonon production (Phonons/(A-Ion)) of the proton beam in the 60–160 MeV energy range, consisting of Al_2_O_3_, SiO_2_, TiO_2,_ and ZrO_2_ ion and recoil-induced interactions in bone phantoms, is given in Table 4. Both ion and recoils were evaluated by comparing ceramic biomaterials with bone by multiplying by 10^4^ for the phonon production numbers with recoil to be meaningful. The ion-induced phonon production in the bone phantom was 0.73 × 10^4^ Phonons/(A-Ion) on average. In the six energy ranges of Al_2_O_3_, SiO_2_, TiO_2,_ and ZrO_2_ phantoms, the ion-derived phonon production values were, respectively, 1.05, 0.73, 1.03, and 0.97 × 10^4^ Phonons/(A-Ion) made of biomaterial. The difference in the remaining biomaterials is average, respectively, occurring at 33.3% in ZrO_2_, 40.6% in TiO_2,_ and 43.9% in Al_2_O_3_. As the energy of the proton beam increased, the ion-induced phonon production increased by an average of 10.6% in the bone phantom. This increase in Al_2_O_3_, SiO_2_, TiO_2,_ and ZrO_2_ phantoms, respectively, 11.2%, 9.7%, 9.6%, and 10.8%. In the bone phantom, the phonon production from recoils was realized as 1.35 × 10^4^ Phonons/(A-Ion) on average. Recoil-induced phonon production values of Al_2_O_3_, SiO_2_, TiO_2,_ and ZrO_2_ phantoms in six energy ranges were, respectively, 2.64, 1.95, 2.73, and 2.82 × 10^4^ Phonons/(A-Ion). In ion-derived phonon production, the biomaterial closest to the bone consisted of SiO_2_ biomaterial with an average difference of 44.2%. The difference in the remaining biomaterials is average, respectively. It formed 95.5% in Al_2_O_3_, 102.1% in TiO_2,_ and 108.3% in ZrO_2_. As the energy of the proton beam increased, the phonon production from recoils increased by an average of 10.7% in the bone phantom. This increase in Al_2_O_3_, SiO_2_, TiO_2,_ and ZrO_2_ phantoms, respectively, was 11.9%, 9.8%, 9.6%, and 7.6%.

**Figure 4:**
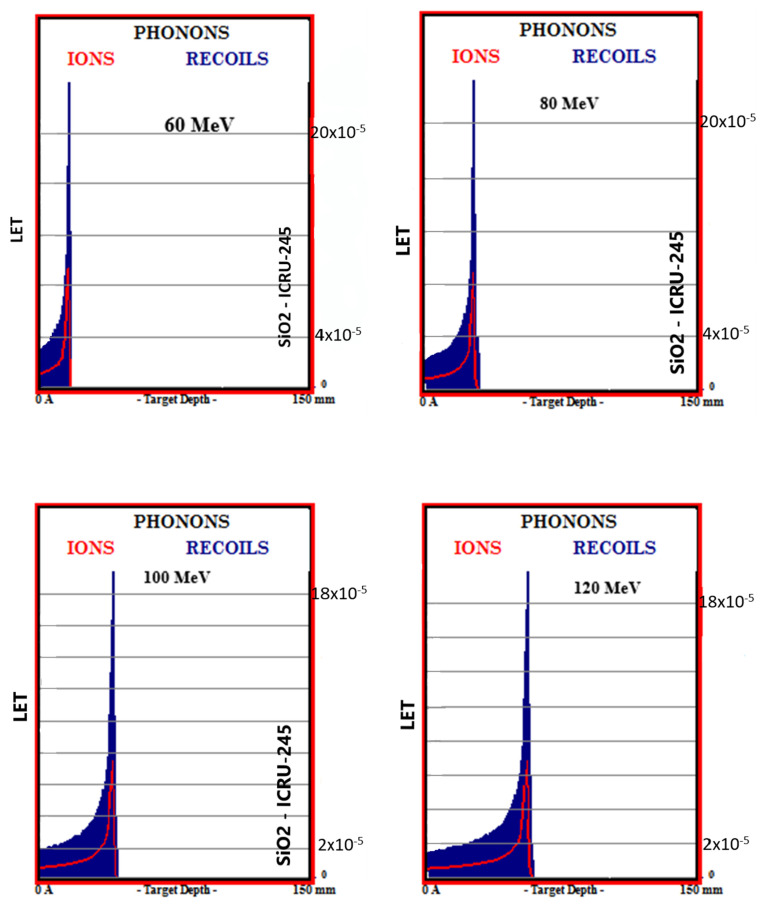

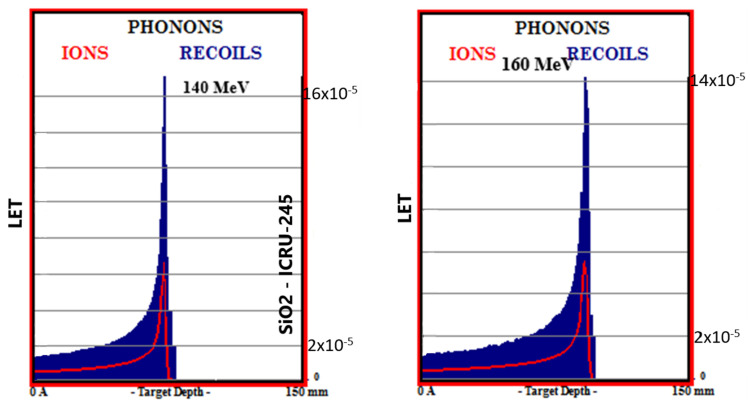
MC TRIM simulation output figures of phonon production in SiO_2_ ceramic biomaterial of a 106-particle proton beam with 60–160 MeV energy.

Simulation outputs of SiO_2_ biomaterial, which is the ceramic biomaterial that gives the closest value to hard tissue in phonon production results are given in Figure 4.

### 3.4. Collision Events

Total collision events (Number/(A-Ion)) caused by the ionization and recoil interactions of the proton beam in the 60–160 MeV energy range with collisions with atoms in Al_2_O_3_, SiO_2_, TiO_2_, ZrO_2,_ and bone phantoms are given in Table 5. With the 20 MeV energy increase in the proton beam, an increase in the values of collision events occurred in bone 13.2%, Al_2_O_3_ 12.6%, SiO_2_ 13.6%, TiO_2_ 13.7%, and ZrO_2_ 13.1%. In total target vacancies, the closest ceramic biomaterial to the bone was Al_2_O_3_ with a 1.2% difference. The differences between the remaining ceramic biomaterials and bone are, respectively, 1.9% in TiO_2_, 11.7% in ZrO_2,_ and 18.4% in SiO_2_. In total target displacements, the closest biomaterial to the bone was Al_2_O_3_ with a difference of 1.8%. The differences between the remaining biomaterials and bone are, respectively; It was 3.4% in TiO_2_, 13.2% in ZrO_2,_ and 19.4% in SiO_2_. In total target replacement collisions, the ceramic biomaterial closest to the bone was Al_2_O_3_.

**Figure 5:**
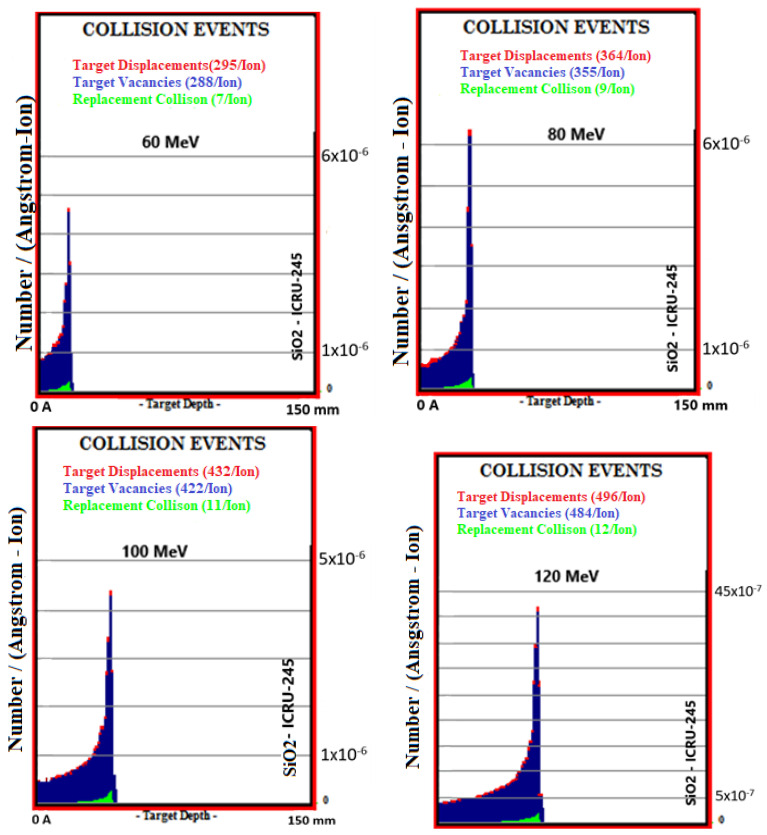

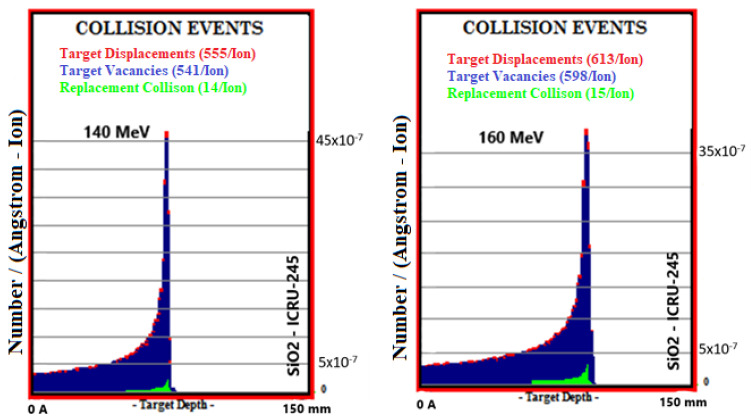
MC TRIM simulation output figures of total collision events values in SiO_2_ ceramic biomaterial of a 106-particle proton beam with 60–160 MeV energy.

Simulation outputs of SiO_2_ biomaterial, which is the ceramic biomaterial that gives the closest value to hard tissue in total collision events values, are given in Figure 5.

### 3.5. Lateral Straggle

**Figure 6:**
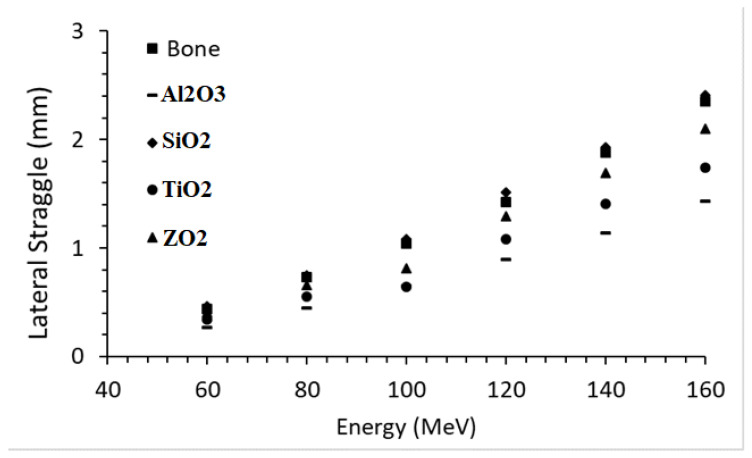
Lateral straggle formed in the beam direction while moving through ceramic and bone biomaterials of the proton beam in the energy range.

The lateral straggle formed in the beam direction as a result of interactions between Al_2_O_3_, SiO_2_, TiO_2_, ZrO_2,_ and bone phantoms of proton beams in the 60–160 MeV energy range is given in Figure 6. The proton beam formed in the bone phantom with an average of 1.310 mm lateral straggle and there was an 81.4% increase between the lowest range and the largest range. While the mean lateral straggle was 1.357 mm in the SiO_2_ phantom, there was an 80.7% increase between the lowest range and the largest range. In the ZrO_2_ phantom, the mean lateral straggle was 1.159 mm, while there was an 80.6% increase between the lowest range and the largest range. In the TiO_2_ phantom, the mean lateral straggle was 0.961 mm, while there was an 80.6% increase between the lowest range and the largest range. While the average lateral straggle was 0.805 mm in the Al_2_O_3_ phantom, there was an increase of 81.1% between the lowest range and the largest range. Ceramic biomaterials closest to the bone in lateral straggle are, respectively; It was found as SiO_2_, ZrO_2_, TiO_2,_ and Al_2_O_3_.

## 4. Discussion

In this study, the Bragg peak range, Bragg peak amplitude, recoils, collision event values, phonon formation, and lateral straggle lengths of the therapeutic energy proton beam were calculated in four different ceramic phantoms and compared with the bone phantom. A proton beam was used in this study, especially since the effect of LET on biological properties and lateral dose distribution may differ between selected ion beam types [31]. The ion type to be selected can be customized by considering these differences and the target tissue with different properties [32]. At this point, it has been a critical issue to investigate the biomaterials closest to the selected hard tissue, i.e., bone. For this reason, bone and four different ceramic biomaterials were selected for the phantom material and compared with bone. As presented in Table 1, the properties of ceramic biomaterials in the MC TRIM simulation system are given. The results obtained with these calculations are compared in Table 2. At the Bragg peak position, the ceramic biomaterial closest to the bone was SiO_2_ with a difference of 10.6%. There is a difference of 47.9% between the atomic density of SiO_2_ and bone biomaterials and 25.4% between their densities. The ceramic biomaterial, which had the biggest difference with bone, was ZrO_2_ with a 53.7% difference. There is a 37.8% difference between the atomic densities of ZrO_2_ and bone biomaterials and a 207% difference between their densities. Thus, it has been observed that the Bragg peak range is largely due to density. Among the selected ceramic biomaterials, it was observed that SiO_2_ was the closest to the bone with a mean Bragg peak amplitude of 3.2%, while the farthest was TiO_2_ with a 56.2% difference. At this point, SiO_2_ was the closest ceramic biomaterial to the bone in Bragg peak location and amplitude. Ionization interactions constitute almost all of the total interactions. Accordingly, the recoil peak is approximately 10^3^ times less than the Bragg peak. Recoil interactions are the main reason for both heavy ion treatment close to critical points, deviations in the direction of the ion beam’s progression within the target, and secondary ion and phonon formations that occur as a result of these two conditions. The mean recoil value closest to the bone was formed in the SiO_2_ ceramic phantom with a difference of 1.7%. It was seen that the biggest contribution to the recoil value was related to the mass percentage of the atom forming the biomaterial, as given in Table 3. Since the percentages of O in bone, Al_2_O_3_ and SiO_2_ biomaterials and Z atom in Ti, and ZrO_2_ biomaterials in TiO_2_ biomaterials are high, the greatest contribution to the recoil’s interaction came from these atoms. These results agreed with the literature [29]. Phonons produced from all interactions of the proton beam in ceramic biomaterials were investigated by considering SRIM MC [29], similar to this study [33]. In particular, the produced phonons were found to be compatible with the literature [29]. SiO_2_ was the closest ceramic biomaterial to bone in ion and recoil-based phonon production. When the remaining biomaterials are evaluated, respectively, they are listed as TiO_2_ and Al_2_O_3_. In experimental studies, diverse types of biomaterials were investigated in experimental studies with the help of heavy ion beams [34]. It is a known fact that the energy coming from the phonons increases the total dose. This increase may also occur at points further away from the intended interaction point. This may cause damage to healthy tissues and may pose a risk of secondary cancer. In this study, ceramic biomaterials were investigated with the help of a simulation system since there was no experimental ion line. Similar properties were investigated using liquid biomaterials in our previous study [35]. In particular, the interaction of ion beams passing through layers and structures with different properties has revealed the importance of choosing the closest biomaterials to biological tissues. These studies have been the subject of research not only in heavy ions but also in conventional beams [36]. The effect of Al_2_O_3_ and ZrO_2_ ceramic biomaterials on the dose was investigated with the help of different ionizing rays and MC [37]. In the collision events presented in Table 5, the ceramic biomaterials that give the closest result to the bone are, respectively, Al_2_O_3_, TiO_2_, ZrO_2,_ and SiO_2_. Therefore, the closest crystal structure to bone was found to be Al_2_O_3_. Considering the lateral scattering values in the selected phantoms, the closest biomaterial to bone was SiO_2_ with a difference of 3.6%. Lateral scattering increased by an average of 0.389 mm for each 20 MeV energy increase in the proton beam in SiO_2_ ceramic biomaterial. This mean increase was 0.382 mm in bone, 0.339 in ZrO_2_, 0.280 in TiO_2_, and 0.232 mm in Al_2_O_3_. Lateral scatter. It is a parameter that should be considered in the treatment of tumors close to critical tissues [38,39,40]. In our previous studies, besides the layered biological structures, the contribution of the biomaterials placed between the biological structures to the lateral scattering was investigated [3]. The effects of therapeutic biomaterials on lateral scattering have been investigated, especially in organs such as the brain, where critical tissues are concentrated [40]. In addition, lateral scattering has also been evaluated in terms of different heavy ion species in similar biomaterials [2]. It was found to be compatible with this study. Additive manufacturing, also known as 3D printing, is a relatively new technique for creating scaffolds with customizable pore shapes and sizes [41]. Chuck Hall introduced the concept in 1986, and it has since revolutionized tissue engineering [41]. By using medical imaging data, personalized scaffolds can be produced to accurately fill injury sites and potentially reconstruct entire organs with complex microstructures [41]. Recent studies highlight the promising applications of additive manufacturing in creating functional tissue scaffolds [42,43]. Bioprinting is an innovative method introduced in the last decade for creating living tissues. It utilizes bioprinters to distribute cells and extracellular matrix components with precision. This exclusive advantage of 3D bioprinting is highlighted in recent research by Santoni et al. [42]. Additive manufacturing methods such as 3D printing offer precise control over pore shape, size, and distribution in scaffolds, allowing easy adjustment of their physicochemical properties [41]. Studies by Jin et al. [44] and Büyük et al. [45] have explored how modifying strand distance, diameter, and stacking pattern in 3D-printed bone scaffolds can regulate their degradation.

## 5. Conclusions

In this study, the interaction of a proton beam with bioceramics, ionization, rebound, collision phenomenon, phonon production, and lateral scattering was investigated with the help of TRIM MC. Phonon production resulting from ionization and recoil interactions was investigated and found to be compatible with the literature. The change properties in the crystal structure of bioceramics, when bombarded with a proton beam, were investigated with collision event parameters and presented as an innovation in this study. The main innovation of this study is to examine the reactions of ceramic biomaterials, which are bone-equivalent biomaterials, to proton therapy in both electron and atomic dimensions. In addition, the results of lateral scattering in the proton beam as a result of ionization and recoil interactions are examined and discussed with similar studies in the literature. As a result of the obtained results and studies in the literature, ceramic biomaterials, which are thought to be close to bone tissue, were compared. The disparity in the Bragg peak position of the proton beam within ceramic biomaterials is believed to stem from the presence of minute air spaces found in biological materials such as bone, as well as disparities in crystal structures. Furthermore, it has been observed that denser materials, despite having a density similar to that of bone, are likely to exhibit these distinctions. Since the energy and particle number of the sent proton beam is at the treatment level, it has been evaluated that it will not cause physical damage such as a fracture in the bone or in the ceramic biomaterial. So much so that, with the beams with proton beam properties in this study, treatments are still ongoing today. However, the main innovation of this study is how many atoms in each ceramic biomaterial are removed from the crystal structure and what interactions result in energy depletion. It is recommended that these calculations be made for different heavy ions as well. Considering the importance of investigating ceramic biomaterials closest to the bone tissue in radiotherapy, different ceramic biomaterials were evaluated.

## Figures and Tables

**Table 1:** Atomic and mass percent, atomic number, and mass density values of ceramic biomaterials selected from the MC TRIM system library [27].

Biomaterials	Atomic Percent	Mass Percent	Atomic Number Density atoms/cm^3^	Mass Density
Bone	H 52.7; O 21.3; C 19.2; N 1.6; S 0.051; Ca 3.05; P 1.87; Mg 0.068	H 6.39; O 41; C 27.7; N 2.69; S 0.2; Ca 14.7; P 6.99; Mg 0.199	13.39	1.85
Al_2_O_3_	O 60; Al 39.96	Al 52.92; O 47.04	11.75	3.98
SiO_2_	O 66.6; Si 33.3	O 53.2; Si 46.7	6.98	2.32
TiO_2_	O 66.6; Ti 33.3	O 40; Ti 59.9	9.56	4.32
ZrO_2_	O 66.6; Zr 33.3	O 25.9; Zr 74	8.33	5.68

**Table 2:** Bragg peak positions and Bragg peak amplitudes formed by the proton beam in phantoms formed from ceramic biomaterials with the help of MC TRIM system.

Energy (MeV)	Bragg Peak Location (cm)					Bragg Peak Amplitude (eV/A)				
	Al_2_O_3_	SiO_2_	TiO_2_	ZrO_2_	Bone	Al_2_O_3_	SiO_2_	TiO_2_	ZrO_2_	Bone
60	0.9	1.7	1.1	0.9	1.8	0.903	0.845	0.859	1.018	0.888
80	1.7	2.7	1.7	1.4	3.0	1.063	0.884	1.440	1.202	0.828
100	2.4	3.9	2.4	2.1	4.5	1.169	0.656	1.198	0.972	0.670
120	3.3	5.4	3.3	2.9	6.2	1.101	0.651	1.138	0.993	0.687
140	4.2	7.2	4.4	3.6	8.0	0.899	0.679	0.949	1.117	0.590
160	5.4	9.0	5.4	4.5	10.1	1.023	0.644	1.016	0.985	0.562

**Table 3:** The total recoil value in the ceramic biomaterials of the proton beam in the energy range and the percentage contribution of the atoms forming the ceramic biomaterials to this amplitude.

Biomaterials	Energy (MeV)	Total Recoil (eV/(A-Ion)	Contributions to Recoils of Atoms (%)											
			O	Al	Si	Ti	Zr	H	C	N	Mg	P	S	Ca
Bone	60	0.350	33.6					26.3	22.3	2.0	0.4	5.1	0.2	10.1
	80	0.308	31.9					27.6	22.3	2.3	0.2	5.1	0.2	10.4
	100	0.276	30.8					30.3	22.1	2.0	0.1	4.5	0.1	10.1
	120	0.259	33.7					26.4	22.7	2.3	0.2	4.5	0.2	10.0
	140	0.227	33.1					26.4	22.5	2.5	0.1	5.2	0.1	10.1
	160	0.198	33.3					26.4	22.3	2.3	0.2	5.2	0.1	10.2
Al_2_O_3_	60	0.297	51.1	48.9										
	80	0.374	51.0	49.0										
	100	0.399	50.4	49.6										
	120	0.378	51.0	49.0										
	140	0.293	50.5	49.5										
	160	0.329	51.5	48.5										
SiO_2_	60	0.326	53.5		46.5									
	80	0.325	53.3		46.7									
	100	0.261	53.3		46.7									
	120	0.239	51.5		48.5									
	140	0.236	54.5		45.5									
	160	0.205	54.9		45.1									
TiO_2_	60	0.322	48.9			51.1								
	80	0.391	47.1			52.9								
	100	0.383	46.7			53.3								
	120	0.376	47.8			52.2								
	140	0.331	47.5			52.5								
	160	0.311	46.9			53.1								
ZrO_2_	60	0.381	36.5				63.5							
	80	0.357	34.5				65.5							
	100	0.354	37.3				62.7							
	120	0.353	35.9				64.1							
	140	0.331	34.7				65.3							
	160	0.299	37.7				62.3							

**Table 4:** Phonon production consisting of interactions originating from ions and recoils in the ceramic biomaterial phantoms of the proton beam in the energy range.

Energy (MeV)	Phonon by Ion (Phonons/(A-Ion))					Phonon by Recoils (Phonons/(A-Ion))				
	Al_2_O_3_	SiO_2_	TiO_2_	ZrO_2_	Bone	Al_2_O_3_	SiO_2_	TiO_2_	ZrO_2_	Bone
60	0.945	0.914	0.971	1.091	0.937	2.354	2.435	2.549	3.146	1.741
80	1.156	0.881	1.167	0.904	0.853	2.894	2.329	3.092	2.761	1.571
100	1.201	0.734	1.121	1.051	0.766	3.018	1.961	2.991	2.961	1.412
120	1.135	0.676	1.062	1.064	0.704	2.856	1.804	2.844	2.971	1.302
140	0.915	0.656	0.975	0.875	0.592	2.287	1.731	2.581	2.612	1.101
160	0.961	0.542	0.874	0.862	0.535	2.454	1.441	2.342	2.457	0.989

**Table 5:** Total collision events values formed by proton beam in phantoms formed from ceramic biomaterials with the help of MC TRIM system.

Collision Events	Biomaterials	Energy (MeV)					
		60	80	100	120	140	160
Total Target Vacancies (Number/(A-Ion))	Bone	234	291	344	393	443	489
	Al_2_O_3_	238	294	348	398	449	493
	SiO_2_	288	355	422	484	541	598
	TiO_2_	239	296	352	400	451	498
	ZrO_2_	265	329	386	445	504	556
Total Target Displacements (Number/(A-Ion))	Bone	237	294	348	398	448	495
	Al_2_O_3_	242	299	354	406	458	503
	SiO_2_	295	364	432	496	555	613
	TiO_2_	246	304	361	411	463	512
	ZrO_2_	272	339	398	458	518	572
Total Target Replacement Collisions (Number/(A-Ion))	Bone	3	4	4	5	5	6
	Al_2_O_3_	5	6	7	8	9	9
	SiO_2_	7	9	11	12	14	15
	TiO_2_	6	8	9	11	12	13
	ZrO_2_	8	10	11	13	15	16

## Data Availability

Data sharing not applicable.
